# Online community management as social network design: testing for the signature of management activities in online communities

**DOI:** 10.1007/s41109-017-0049-9

**Published:** 2017-08-30

**Authors:** Alberto Cottica, Guy Melançon, Benjamin Renoust

**Affiliations:** 1 University of Alicante, Alicante, Spain; 2 Edgeryders, Tallinn, Estonia; 3 Université de Bordeaux, Talence, France; 4 CNRS UMR 5800 LaBRI, Bordeaux, France; 5 National Institute of Informatics, Tokyo, Japan; 6 CNRS UMI 3527, JFLI, Tokyo, Japan

**Keywords:** Collective intelligence, Online communities, Network structure

## Abstract

Online communities are used across several fields of human activities, as environments for large-scale collaboration. Most successful ones employ professionals, sometimes called “community managers” or “moderators”, for tasks including onboarding new participants, mediating conflict, and policing unwanted behaviour. Network scientists routinely model interaction across participants in online communities as social networks. We interpret the activity of community managers as (social) network design: they take action oriented at shaping the network of interactions in a way conducive to their community’s goals. It follows that, if such action is successful, we should be able to detect its signature in the network itself.

Growing networks where links are allocated by a preferential attachment mechanism are known to converge to networks displaying a power law degree distribution. Growth and preferential attachment are both reasonable first-approximation assumptions to describe interaction networks in online communities. Our main hypothesis is that managed online communities are characterised by in-degree distributions that deviate from the power law form; such deviation constitutes the signature of successful community management. Our secondary hypothesis is that said deviation happens in a predictable way, once community management practices are accounted for. If true, these hypotheses would give us a simple test for the effectiveness of community management practices.

We investigate the issue using (1) empirical data on three small online communities and (2) a computer model that simulates a widely used community management activity called *onboarding*. We find that onboarding produces in-degree distributions that systematically deviate from power law behaviour for low-values of the in-degree; we then explore the implications and possible applications of the finding.

## Introduction

Organizations running online communities typically employ community managers, tasked with encouraging participation and resolving conflict (Rheingold 1993). These are participants, typically in small numbers (one or two members in the smaller communities) who recognise some central command, and carry out its directives. We shall henceforth call such directives *policies*.

Putting in place policies for online communities is costly, in terms of community managers recruitment and training, and software tools. This raises the question of what benefits organisations running online communities expect from policies; and why they choose certain policies, and not others. In what follows we outline and briefly discuss the set of assumptions that underpin our investigation.

We model online communities as social networks of interactions across participants. An implicit assumption in our work is that the topology of the interaction network of online communities affects their ability to reach their objectives (that can be formed in terms of the maximization of some objective function^1^, see for instance (Tapscott and Williams 2008; Slegg 2014).

Community managers may thus derive a course of actions to alter the interaction patterns of their communities, so as to favor and support the achievement of the community’s objectives.

The actions can be encoded as a set of simple instructions for community managers to execute. Computer scientists might think of such instructions as algorithms; economists call them mechanisms; professional online community managers call them policies. In this paper we use this third term.

All this implies that the decision to deploy a particular policy on an online community is a network design exercise. An organisation decides to employ a community manager to shape the interaction network of its community in a way that helps its own ultimate goals. And yet, interaction networks in online communities cannot simply be designed; they are the result of many independent decisions, made by individuals who do not respond to the organization’s command structure. An online community management policy is then best understood as an attempt to “influence” emergent social dynamics; to use a more synthetic expression, it can be best understood as the attempt to design for emergence. Its paradoxical nature is at the heart of its appeal.

We are interested in detecting the mathematical signature of specific policies in the network topology. We consider a simple policy called *onboarding* (Rheingold 1993; Shirky 2008). As a new participant becomes active (e.g. by posting her first post), community managers are instructed to leave her a comment that contains (a) positive feedback and (b) suggestions to engage with other participants that she might share interests with.

We model online conversations as social networks, and look for the effect of onboarding on the topology of those networks. We proceed as follows:

1. We initially examine data from three small online communities. Only two of them deploy a policy of onboarding. We observe that, indeed, the shape of the degree distribution of these two differs from that of the third.
2. We propose an experiment protocol to determine whether onboarding policies can explain the differences observed between the degree distributions of the first two online communities and that of the third one.
3. Based on a generalized preferential attachment model (Dorogovtsev and Mendes 2002), we simulate the growth of online communities. Variants to the model cover the relevant cases: the absence of onboarding policies and their presence, with varying degrees of effectiveness.
4. We run the experiment protocol against the degree distributions generated by the computer model, and discuss its results.

“Related works” section briefly examines the two strands of literature that we mostly draw upon. “Materials and methods” section presents some data from real-world online communities; it then proceeds to describe our main experiment, a computer simulation of interaction in online communities with and without onboarding. “Results” section presents the experiment’s results. “Discussion and conclusions” section discusses them.

## Related works

The extraordinary successes of online communities in deploying large-scale, decentralized projects has led many scholars to conjecture that online communities exhibit emergent behavior, and called such behavior collective intelligence, after an influential book by Pierre Lévy (1997). This name was adopted by a research community that aims at providing tools for better collective sense- and decision making such as argument maps (representations of the logical structure of a debate, with all redundancy eliminated) (Shum 2003) and attention-mediation metrics (indicators that signal what, in an online debate, is worthiest reading and responding to. The number of Likes on Facebook is one such metric) (Klein 2012).

Alongside with positive studies, scholars have researched the normative aspects of online community management. The monography by (Kraut et al. 2012) confirms the importance of online community management practices, and even proposes a categorization and critical look at existing practices. Others have tried to systematic approaches to community build (Diplaris et al. 2011) and produce technological innovation to support it (Shum 2003; De Liddo et al. 2012). These tools are meant to facilitate and encourage participation to online communities, to make it easier for individuals to extract knowledge from them.

Starting in the 2000s, online communities became the object of another line of enquiry, stemming from network science. Network representation of relationships across groups of humans has yielded considerable insights in social sciences since the work of the sociometrists in the 1930s, and continues to do so; phenomena like effective spread of information, innovation adoption, and brokerage have all been addressed in a network perspective (Borgatti et al. 2009; Burt 2009). As new datasets encoding human interaction became available, many online communities came to be represented as social networks. This was the case for social networking sites, like Facebook (Lewis et al. 2008; Nick 2013); microblogging platform like Twitter (Kunegis et al. 2013; Java et al. 2007; Hodas and Lerman 2014); news-sharing services like Digg (Hodas and Lerman 2014); collaborative editing projects like Wikipedia (Laniado et al. 2011); discussion forums like the Java forum (Zhang et al. 2007); and bug reporting services for software developers like Bugzilla (Zanetti et al. 2012). Generally, such networks represent participants as nodes. Edges represent a relationship or interaction. The nature of interaction varies across online communities: one edge can stand for friendship for Facebook; follower-followed relationship, retweet or mention in Twitter; vote or comment in Digg and the Java forum; talk in Wikipedia; comment in Bugzilla.

In contrast to collective intelligence scholars, network scientists typically do not address the issue of community management, and treat social networks drawn from online interaction as fully emergent. In this paper, we employ a network approach to investigate the issue of whether the work of community managers leaves a footprint detectable by quantitative analysis. To our knowledge, no other work attempted this investigation. In particular, we exploit a result from the theory of evolving networks, from seminal work by Barabási and Albert (1999) showing that the assumption of growth and preferential attachment, when taken together, result in a network whose degree distribution converges to a power law (Barabasi 2005; Barabási et al. 1999). The model was later generalized in various ways and tested across a broad range of networks, including social networks (Dorogovtsev and Mendes 2002).

We use this generalized model as a baseline. We acknowledge that there are real-world human communication networks that do not appear to have been generated by it (see for example (Leskovec and Horvitz 2008)). In very large social networks, for example, limitations to human cognition as expressed by Dunbar numbers might truncate the distribution.

The baseline model implies that the in-degree distribution of the interaction network in an online community follows a power law by default. The action of online community managers, as they attempt to further the goals of the organisation that runs the online community, will result in its degree distribution deviating from the baseline power law in predictable ways. Such deviation can be interpreted as the signature that the policy is working well.

The most important difficulty with this method is the absence of a counterfactual: if a policy is enacted in the online community, the baseline degree distribution corresponding to the absence of the policy is not observable, and viceversa. This rules out a direct proof that the policy “works”. Hence our choice to combine empirical data and computer simulations.

In a previous paper (Cottica et al. 2016), we test whether power law models are a good fit for the untransformed in-degree distributions of interaction networks in online communities. The approach presented in this paper is more general in that we transform the in-degree distributions before applying the same test. This is meant to take on board explicitly the node attractiveness parameter mentioned in (Dorogovtsev and Mendes 2002).

## Materials and methods

In this section we introduce the empirical data, the experiment protocol and the simulation model we use in the experiment.

### Empirical data

We examine data from three real-world online communities. We obtained the data from the organisations managing them; in fact, one of the authors is directly involved in two of them, Edgeryders and Matera 2019. The three are roughly comparable in size; all are used by practitioners and interested citizens to publicly discuss issues that have a collective dimension; arise around a shared interest rather than personal ties. The last point is important, since “topical” and “social” online interaction patterns have been shown to be different (Grabowicz et al. 2013).

Online communities are modelled as interaction networks, in which nodes are registered users and edges represent comments. The presence of an edge from Alice to Bob indicates that Alice has commented content authored by Bob at least once. The resulting graphs are directed (“Alice comments Bob” is not equivalent to “Bob comments Alice”) and weighted (Alice can write multiple comments to Bob’s content; the edge’s weight is equal to the number of comments written). Table 1 presents some descriptive statistics about them.

**Table 1:** Comparing interaction networks of the three online communities and testing for goodness-of-fit of power functions to degree distributions

	Innovatori PA	Edgeryders	Matera2019
Policy	*“no special policy”*	*“onboard new users”*	*“onboard new users”*
In existence since	December 2008	October 2011	March 2013
Accounts created	10,815	2419	512
Active participants (nodes)	619	596	198
Number of edges (weighted)	1241	4073	883
Average distance	3.77	2.34	2.51
Maximum degree	155	238	46
Average degree	2.033	6.798	4.454
Goodness-of-fit for *k*≥1			
exponent	1.611	1.477	1.606
*p*-value	0.21	0.00 (reject)	0.00 (reject)
Goodness-of-fit for *k*≥*k*_min_			
*k*_min_	2	5	6
exponent	1.834	2.250	2.817
*p*-value	0.76	0.45	0.94

“Exponent” refers to the power law’s scaling parameter. “*p*-value” to the result of the test that the degree distribution of the community was generated by a power law with that exponent

- InnovatoriPA^2^ is a community of (mostly) Italian civil servants discussing how to introduce and foster innovation in the public sector. It does not employ any special onboarding or moderation policy.
- Edgeryders^3^ is a community of (mostly) European citizens, discussing public policy issues from the perspective of grassroot activism and social innovation. It enacts the onboarding of new members policy.
- Matera 2019^4^ is a community of (mostly) citizens of the Italian city of Matera and the surrounding region, discussing the city’s policies. It, too, enacts an onboarding policy. The two policies are exactly the same; Matera 2019 has modelled its community management policies on those of Edgeryders.

**Fig. 1:**
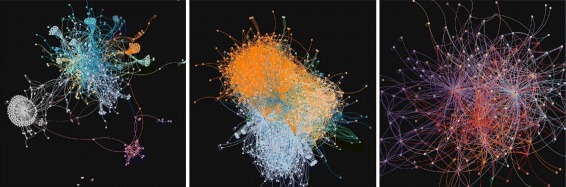
Interaction networks of three small online communities. Innovatori PA (*left*) does not have an onboarding policy in place, whereas the two others do (Edgeryders: *center*, Matera: *right*)

The communities are modeled as interaction networks (summarized in Table 1) in which nodes are users and edges represent directed comments from *A* to *B*, weighted by the number of comments written. A glance at their respective visualizations (Fig. 1) suggests that the networks of the three communities have very different topologies. Innovatori PA displays more obviously visible hubs than the other two.

#### Testing goodness-of-fit

**Fig. 2:**
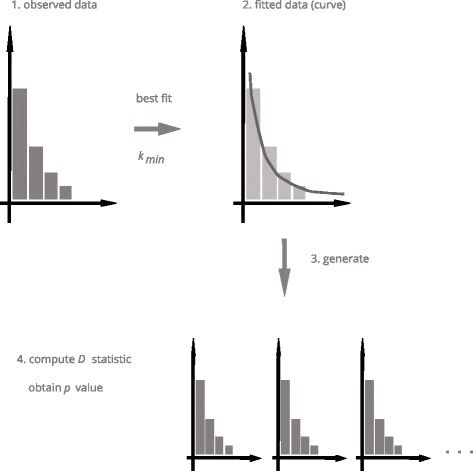
The basic experiment workflow. After fitting the observed data, artificial datasets are generated according to the fit function. The null hypothesis is then accepted or rejected based on the *p*-value derived from the *D* statistic

We fitted power laws in-degree distributions of these three online communities, as of early December 2014. Next, we tested the hypothesis that these in-degree distributions follow a power law, as predicted by (Dorogovtsev and Mendes 2002). See Fig. 2 illustrating the computation workflow.

To do so, we first fitted power functions to the entire support of each in-degree distribution. We emphasize in-degree, as opposed to out-degree, because directedness is implicit in the idea of preferential attachment, and because the in-degree distribution is the one to follow a power law in online conversation networks (Dorogovtsev and Mendes 2002).

**Fig. 3:**
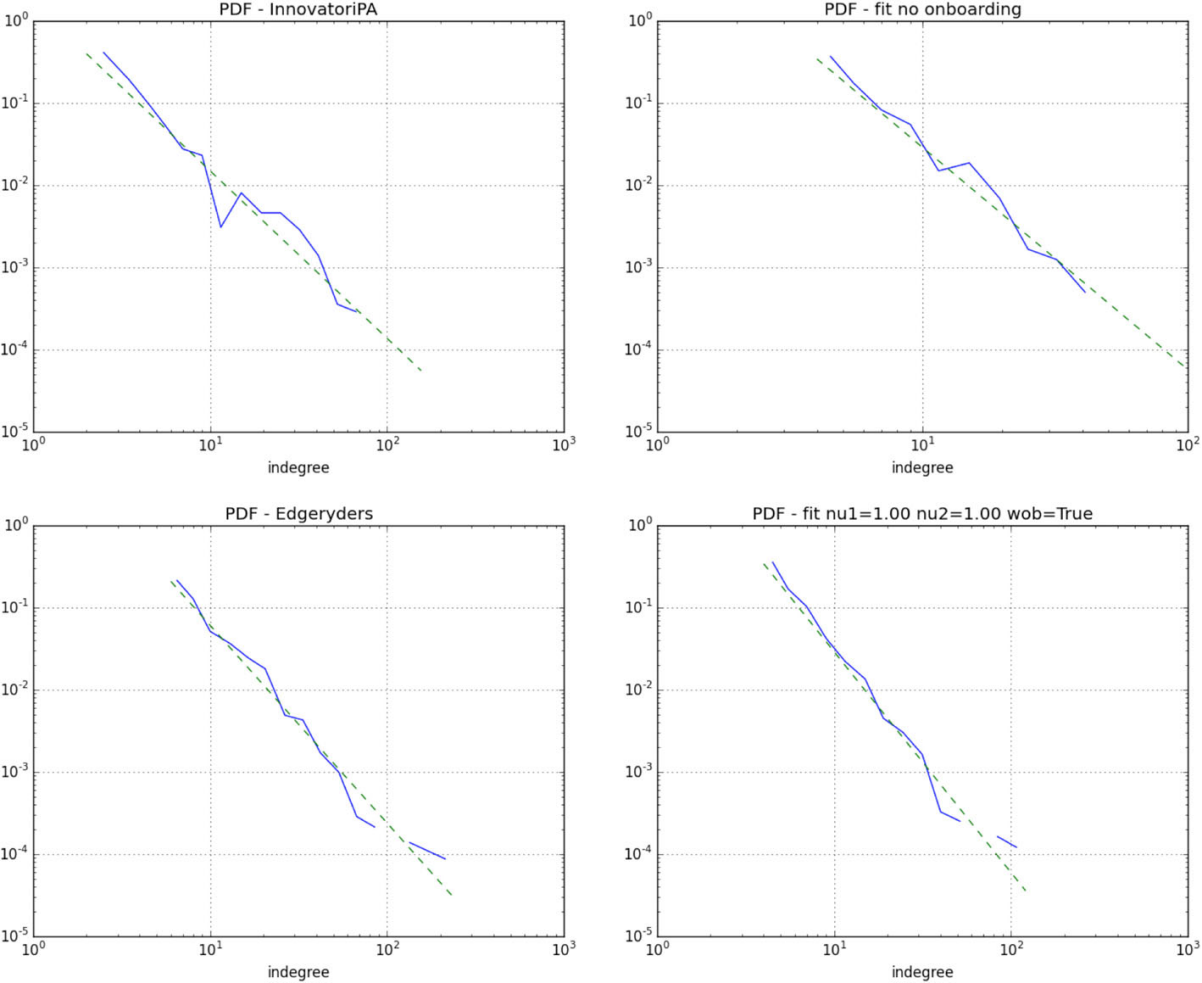
(log - log) Probability density function from the transformed degree distributions. (*top left*) The Innovatori PA network without onboarding policy in place versus (*top right*) a simulated network with preferential attachment and no onboarding. (*bottom left*) The Edgeryders network with onboarding and preferential attachment versus (*bottom right*) a simulated network with preferential attachment and fully effective onboarding (*ν*_1_=*ν*_2_=1)

We next fitted power functions to the right tail of each in-degree distribution, *i.e.* for any degree *k*(*n*)≥*k*_min_, where *k*_min_ is the in-degree that minimizes the Kolmogorov-Smirnov distance (hereafter denoted as *D*) between the fitted function and the data with in-degree *k*(*n*)≥*k*_min_. Figure 3 shows the similarity between the in-degree distributions of the interaction networks of real-life and simulated online communities, with and without onboarding.

Finally, we ran goodness-of-fit tests for each in-degree distribution and for fitted power functions. The method we followed throughout the paper is borrowed from Clauset et al. (2009). In the rest of this section we briefly describe it.

We start from a null hypothesis that the observed distribution is generated by a power function with exponent *α*, on the domain *k*≥*k*_*min*_. We denote by *D* the Kolmogorov-Smirnov distance between the observed distribution and the power function that best fits it. Next, we use the best fit power function to generate a large number (*N*) of distributions. We now denote *D*_*G*_ the Kolmogorov-Smirnov distance between each of them and its own best fit power function. Finally, we compare *D* with *D*_*G*_ for each of the generated distribution.

Such comparison is summarised in a *p*-value. This *p*-value counts the number of times when *D*_*G*_>*D* over *N*. A *p*-value close to 1 indicates that the power function is a good fit for the data: hence the null hypothesis is not rejected. A *p*-value close to zero indicate that the power function is a bad fit for the data, and rejects the null hypothesis. The rejection value is set, conservatively, at 0.1.

The previously described computation is conducted in a systematic way. The observed data is fitted twice, first over the whole in-degree distribution (that is, over the interval *k*≥1), and then over the interval *k*≥*k*_min_. The goodness-of-fit procedure is then ran on both fitted power functions.

#### Results on empirical data

Results are summarised in Table 1. As we consider the interval *k*≥1, we find that the in-degree distribution of the Innovatori PA network – the unmoderated one – is consistent with the expected behavior of an evolving network with preferential attachment. We cannot reject the null hypothesis that it was generated by a power law. For other two communities, both with onboarding policies, the null hypothesis is strongly rejected. On the other hand, when we consider only the tail of the degree distributions, i.e. *k*≥*k*_min_, all three communities display a behavior that is consistent of a setting with preferential attachment.

These results are consistent with the objectives of the onboarding policy, consisting in helping newcomers find their way around a community that they don’t know yet. A successfully onboarded new user will generally have some extra interaction with existing active members. All things being equal, we can expect extra edges to appear in the network, and interfere with the in-degree distribution that would appear in the absence of onboarding – explaining the non-power law distribution of Edgeryders and Matera2019. Extra edges target mostly low connectivity nodes: onboarding targets newcomers, and focuses on helping them through the first few successful interactions. Highly active (therefore highly connected) members do not need to be onboarded. This may explain why all three communities display power law behavior in the upper tail of their in-degree distributions, regardless of onboarding.

The difference observed between the two communities with onboarding policies and the one without might be caused not by the policy itself, but by some other unobserved variable. For example, variations in user experience design choices are associated to different (network) patterns of inter-user communication in (Hodas and Lerman 2014). Cultural differences across the different user bases could also be playing a role. The available evidence is compatible with the hypothesis that onboarding policies in online communities leave a signature in the in-degree distribution of their interaction networks, but it cannot prove that hypothesis.

### Experiment protocol

To explore the issue further, we generate and compare computer simulations of interaction networks in online communities that are identical except for the presence and effectiveness of onboarding policies. In this way, we isolate the effect, on the interaction network, of onboarding from that of any other effect that might be at work in the real world. The mechanics of the model is described in “The simulation model” section.

We proceed as follows.

First, we simulate the evolution of the interaction network of a large number of online communities. We divide them into a control group (no onboarding policy) and a treatment group (presence of onboarding policy). Specifically, we simulate the evolution of the interaction network of:

- One hundred communities with no onboarding policy. These will constitute the control group of our simulated communities.
- One hundred communities for each couple of values of *ν*_1_ and *ν*_2_, with *ν*_1_,*ν*_2_∈{0.0, 0.2, 0.4, 0.6, 0.8, 1.0}. These will constitute our treatment groups.
- For each of these networks, we compute the in-degree distribution.

Next, we define the following hypotheses.

- Let *C* be the network of interaction in an online community. Denote the in-degree of node *n* in the network by *k*(*n*). Let *F* be the best-fit power-law model for the distribution:
  1 $$ q(k) = k + mA  $$
  where *k* is the in-degree distribution of *C*, *m* the number of nodes that join the network at each timestep and *A* a node attractiveness parameter.
- *Hypothesis 1*. The distribution of *q*(*k*) is generated by *F* for any *k*>1.
- *Hypothesis 2*. The distribution of *q*(*k*) is generated by *F* for any *k*≥*k*_min_, where *k*_min_ is the in-degree that minimizes the Kolmogorov-Smirnov distance between the fitted function and the data over *k*≥*k*_min_.

Hypotheses 1 and 2 are similar in scope, but different in strength. Hypothesis 1 rests on the more restrictive condition that the in-degree distribution is a good fit for a power function over its whole domain; Hypothesis 2 needs for the distribution only to be a good fit for a power function over its upper tail. This makes Hypothesis 2 much harder to reject. For example, for Edgeryders and Matera2019, Hypothesis 1 is rejected, whereas Hypothesis 2 is not rejected.

Both hypotheses are based on the asymptotic form taken by the stationary in-degree distribution of networks growing by preferential attachments in (Dorogovtsev and Mendes 2002). The result holds even if preferential attachment is not the sole mode of network evolution, and for any edge sources.

The exact formulation for Hypothesis 1 in (Dorogovtsev and Mendes 2002) is *k*>>1, which we approximate with *k*>1, because onboarding only targets newcomers to an online community, therefore low-degree nodes in the network. In other words, onboarding’s influence on the goodness-of-fit of the transformed in-degree distribution to the power law model is strongest on its lower tail.

Finally, we test Hypothesis 1 and 2 on each of the 3700 in-degree distributions generated. We do this using the goodness-of-fit tests proposed by Clauset et al. (2009) and illustrated in detail in the Appendix. We expect to obtain the following:

- In the control group, both Hypothesis 1 and Hypothesis 2 are true.
- In the treatment group with fully effective onboarding Hypothesis 1 is false and Hypothesis 2 is true.
- In the intermediate situations of partially ineffective onboarding, Hypothesis 1 can be true or false, according to the value of *ν*_1_ and *ν*_2_. Hypothesis 2 is true.

Disproving Hypotheses 1 and 2 implies that, in the context of the model, the micro-level behaviour prescribed by the onboarding policy onto the community manager gives rise to an in-degree distribution that is no longer power law-shaped. The real-world implications of such a result are discussed in “Discussion and conclusions” section.

### The simulation model

Our computer model simulates the growth of an interaction network in an online community with and without onboarding. It follows closely the practices of real-world online community management as we know them, for example as reported in the Edgeryders and Matera 2019 online communities. The purpose of this is to check what effect this micro behaviour has on the network and its degree distribution.

#### Without onboarding

We use the model without onboarding to generate the networks in our control group. The mechanism used for the network to grow is based on preferential attachment, consistently with the Barabási-Albert tradition. We follow the more general formulation of Dorogovtsev and Mendes (2002).

- A (directed) network is initialized, consisting of two reciprocally connected nodes *u*,*v* (thus comprising two directed links *u*→*v*,*v*→*u*).
- At each time step,
  - one new node – representing a participant in the online community – appears in the network.
  - *m* new edges – representing comments – appear in the network. The source of each edge is drawn at random from the uniform distribution of the existing nodes.

This represents a departure from (Dorogovtsev and Mendes 2002), where edge sources are assumed to be unspecified. We need to specify edge sources in order to conform to the data model of the network analysis software we are using; this, however, does not have any analytical implications, as both (Dorogovtsev and Mendes 2002) and we focus on the in-degree distribution.

Its target is chosen according to the following rule: the probability that the new edge points to node *s* is proportional to *k*(*s*)+*A* where *A* is a parameter representing additional attractiveness of the node.

#### With onboarding

We use a variant of the above model that includes onboarding to generate the networks in our treatment group. The variant consists simply of the model without onboarding, to which further steps are added as follows.

- At each timestep, in addition to the *m* edges mentioned above, one additional edge is directed towards the newcomer node. This is meant to represent the community manager’s onboarding action described in “Introduction” section.
- At each timestep, with probability *ν*_1_, one edge is added. Its source is the newcomer node; its target is chosen according to the following rule: the probability that the new edge points to node *s* is proportional to *k*(*s*)+*A* where *A* is a parameter representing additional attractiveness of the node. This is meant to represent the newcomer’s reaction to the community manager’s onboarding activity; as a result of the latter, the newcomer becomes active and reaches out to someone in the community, as advised by the community manager. We assume that community managers will normally incline to point newcomers to existing users who are reputed to be interesting conversationalists, and that the characteristic of being interesting conversationalists is correlated with node in-degree. Parameter *ν*_1_ can be thought of as representing *onboarding effectiveness*. More skilled community managers will be more persuasive in inducing newcomers to reach out and engage in the conversation taking place in the online community.
- At each timestep, one more edge is added with probability *ν*_2_. Its source is drawn at random from the uniform distribution of the existing nodes; its target is the newcomer node. This represents a successful onboarding outcome: the new participant, by becoming active, has attracted the attention of some existing participant, who has engaged with her. No longer isolated, she is now in conversation. *ν*_2_ can be thought of as representing *community responsiveness*. As it increases, the efforts of newcomers to engage in conversation become more likely to be reciprocated.

## Results

Following the protocol outlined above, we evolved 100 networks for each of the 37 variants of the model. For all networks, we set network size to 2000 nodes; *A*=1; and *m*=1. These choices are discussed in the Appendix. Our results are summarized in Table 2.

**Table 2:** Testing for Hypotheses 1 and 2: expectations as declared in “The simulation model” section vs. results

	Expected	Found
Without onboarding	Hypothesis 1: True	Hypothesis 1: 40% True
	Hypothesis 2: True	H2: 85% True
With onboarding (partially effective)	Hypothesis 1: Depends on parameters	Hypothesis 1: > 95% True
	Hypothesis 2: True	Hypothesis 2: 65-80% True
With onboarding (fully effective)	Hypothesis 1: False	Hypothesis 1: False
	Hypothesis 2: True	Hypothesis 2: ∼75% True

### Goodness-of-fit of the power-law model

For each network evolved we computed two best-fit power-law models, one for *k*>1 and the other for *k*≥*k*_min_ where *k*_min_ is the in-degree the minimizes the Kolmogorov-Smirnov distance between the fitted function and the data over *k*≥*k*_min_. On each of these models, we ran a goodness-of-fit test as described in “Experiment protocol” section. This resulted in two distributions of *p*-values for our control group, plus two more for each of our 36 treatment groups. Tables 3 and 4 report descriptive statistics for these distributions.

**Table 3:** Number of rejects (out of 100 runs) for goodness-of-fit tests of power-law models to in-degree distributions of interaction networks in online communities, with no onboarding (control group) and with onboarding

Treatment groups, rejects	*ν*_2_ = 0.0	*ν*_2_ = 0.2	*ν*_2_ = 0.4	*ν*_2_ = 0.6	*ν*_2_ = 0.8	*ν*_2_ = 1
*ν*_1_ = 0.0	99	97	100	99	99	100
*ν*_1_ = 0.2	100	100	99	98	97	98
*ν*_1_ = 0.4	98	98	96	99	100	98
*ν*_1_ = 0.6	96	96	99	99	99	98
*ν*_1_ = 0.8	98	97	98	99	98	98
*ν*_1_ = 1	98	98	100	100	99	98
Control group, rejects: 61						

Power-law models are estimated over all nodes with degree *k*>1

**Table 4:** Treatment groups: average *p*-values for goodness-of-fit tests of power-law models to in-degree distributions of interaction networks in online communities, with no onboarding (control group) and with onboarding

Treatment groups, average *p*-value	*ν*_2_ = 0.0	*ν*_2_ = 0.2	*ν*_2_ = 0.4	*ν*_2_ = 0.6	*ν*_2_ = 0.8	*ν*_2_ = 1
*ν*_1_ = 0.0	0.005	0.010	0.005	0.007	0.006	0.007
*ν*_1_ = 0.2	0.005	0.006	0.009	0.011	0.014	0.012
*ν*_1_ = 0.4	0.009	0.009	0.015	0.005	0.006	0.008
*ν*_1_ = 0.6	0.013	0.012	0.008	0.009	0.010	0.008
*ν*_1_ = 0.8	0.009	0.015	0.012	0.009	0.012	0.009
*ν*_1_ = 1	0.009	0.011	0.009	0.008	0.010	0.013
Control group, average *p*-value: 0.183						

Power-law models are estimated over all nodes with degree *k*>1

From Table 3, we conclude that onboarding seems to have some effect on the goodness-of-fit of the generated data to their respective best-fit power-law models when *k*>1. The effect goes in the direction of reducing the *p*-values and increasing the number of rejects to almost 100%.

It is worth looking at the average *p*-values generated by each combination of *ν*_1_ and *ν*_2_. These are shown in Table 4.

We run *t*-tests of the null hypothesis that the average *p*-value in the control group is equal to the average *p*-values in each of the different treatment groups. This results in a strong rejection of the null for any combination of *ν*_1_ and *ν*_2_ (6.5<*T*<7.5 in all cases). It seems unquestionable that introducing onboarding to an online community has a measurable negative impact on the probability of a power-law model to be a good fit for its interaction network’s in-degree distribution.

When we consider only the upper tail of the the distribution generated by Eq. 1, the effect of introducing onboarding on the goodness-of-fit is much less clear. In Table 5 we show what happens when we choose the scaling range so as to minimize the Kolmogorov-Smirnov distance between the degree distributions themselves and their best-fit power-law models. In the control group, the goodness-of-fit-to-power-law test fails in 13 of the 100 runs. In the treatment groups, rejections vary from 18 to 36, depending on the values of *ν*_1_ and *ν*_2_.

**Table 5:** Treatment groups: number of rejects (out of 100 runs) for goodness-of-fit tests of power-law models to in-degree distributions of interaction networks in online communities, with no onboarding (control group) and with onboarding

Treatment groups, rejects	*ν*_2_ = 0.0	*ν*_2_ = 0.2	*ν*_2_ = 0.4	*ν*_2_ = 0.6	*ν*_2_ = 0.8	*ν*_2_ = 1
*ν*_1_ = 0.0	34	35	35	25	22	28
*ν*_1_ = 0.2	35	24	30	34	29	29
*ν*_1_ = 0.4	28	22	25	34	27	26
*ν*_1_ = 0.6	29	27	18	23	28	19
*ν*_1_ = 0.8	26	27	28	36	32	18
*ν*_1_ = 1	28	28	18	27	21	27
Control group, rejects: 13						

Power-law models are estimated over all observations with *k*≥*k*_min_

Average *p*-values of goodness-of-fit tests when *k*≥*k*_min_ are shown in Table 6. They are all well within the do-not-reject range.

**Table 6:** Treatment groups: average *p*-values for goodness-of-fit tests of power-law models to in-degree distributions of interaction networks in online communities, with no onboarding (control group) and with onboarding

Treatment groups, average *p*-value	*ν*_2_ = 0.0	*ν*_2_ = 0.2	*ν*_2_ = 0.4	*ν*_2_ = 0.6	*ν*_2_ = 0.8	*ν*_2_ = 1
*ν*_1_ = 0.0	0.341	0.341	0.345	0.368	0.411	0.355
*ν*_1_ = 0.2	0.328	0.399	0.364	0.339	0.324	0.381
*ν*_1_ = 0.4	0.382	0.408	0.367	0.341	0.414	0.372
*ν*_1_ = 0.6	0.348	0.372	0.4087	0.381	0.413	0.409
*ν*_1_ = 0.8	0.370	0.383	0.382	0.324	0.359	0.436
*ν*_1_ = 1	0.383	0.401	0.458	0.413	0.4	0.393
Control group, average *p*-value: 0.451						

Power-law models are estimated over all observations with *k*≥*k*_min_

Tables 5 and 6 tell two different stories. Table 5 is unconclusive: in both the control and the treatment groups, we do not reject Hypothesis 2 in the treatment group most of the time, as expected, but must still reject in a relatively large number of cases (13 in the control group, 18-36 in the treatment groups). Table 6 indicates that the average *p*-value in all groups is comfortably within the do-not-reject range, and in this sense behaves entirely according to Hypothesis 2.

### Lower bounds

Our results show a limited, albeit statistically significant, effect of onboarding on the value of *k*_min_, the value of *k* that minimizes the Kolmogorov-Smirnov distance between the data generated by the computer simulation and the best-fit power-law model. Table 7 shows, for each value of *ν*_1_ and *ν*_2_, the average value of *k*_min_, and the result (expressed in *p*-value) of a *t*-test on the null hypothesis that such average value is the same as the corresponding statistics in the control group, against the alternative hypothesis that the former is greater than the latter.

**Table 7:** Average values of *k*_min_ in the control group and in the treatment group by values of *ν*_1_ and *ν*_2_

Treatment groups, average *k*_min_	*ν*_2_ = 0.0	*ν*_2_ = 0.2	*ν*_2_ = 0.4	*ν*_2_ = 0.6	*ν*_2_ = 0.8	*ν*_2_ = 1
*ν*_1_ = 0.0	2.23 (0.006)	2.3 (0.003)	2.38 (0.001)	2.33 (0.001)	2.48 (0.000)	2.34 (0.001)
*ν*_1_ = 0.2	2.42 (0.000)	2.45 (0.000)	2.46 (0.000)	2.36 (0.001)	2.33 (0.001)	3.3 (0.004)
*ν*_1_ = 0.4	2.51 (0.000)	2.68 (0.000)	2.44 (0.000)	2.27 (0.007)	2.49 (0.000)	2.59 (0.000)
*ν*_1_ = 0.6	2.42 (0.000)	2.35 (0.001)	2.63 (0.000)	2.5 (0.000)	2.53 (0.000)	2.65 (0.000)
*ν*_1_ = 0.8	2.44 (0.000)	2.54 (0.000)	2.49 (0.000)	2.34 (0.001)	2.26 (0.007)	2.56 (0.000)
*ν*_1_ = 1	2.55 (0.000)	2.52 (0.000)	2.66 (0.000)	2.58 (0.000)	2.5 (0.000)	2.49 (0.000)
Control group, average *k*_min_: 1.87						

The number in parenthesis is the *p*-value associated to a *t*-test that *k*_min_(*t* *r* *e* *a* *t* *m* *e* *n* *t*)=*k*_min_(*c* *o* *n* *t* *r* *o* *l*)

**Fig. 4:**
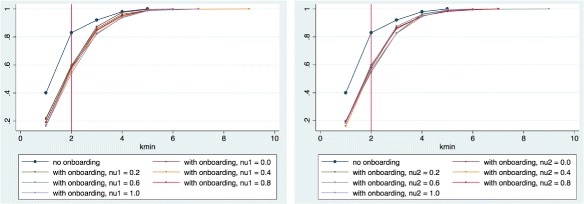
Cumulate Density Functions of the average value of *k*_min_ that minimizes the Kolmogorov-Smirnov distance between the in-degree distribution of each interaction network and its best-fit power-law model in the control and treatment groups. Twenty percent of the networks evolved without onboarding (*dark blue*) have degree distributions that test negatively for H1. When onboarding is introduced, that percentage rises to between 50 and 90%. On the *left*, the treatment group interaction networks have been grouped according to the value taken by *ν*_1_; on the *right*, they have been grouped according to the value taken by *ν*_2_

A glance at Fig. 4 shows that over 80% of the in-degree distributions from interaction networks in the control group, vis-a-vis only 50 to 60% of those in the treatment group, fit a power-law model best for *k*_min_≤2. Within the treatment group, no significant variability seems to be associated to the increase of either *ν*_1_ or *ν*_2_.

### Exponents

We find that introducing onboarding to an online community has a positive and significant effect on the value of the exponent of the best-fit power-law model for the in-degree distribution of its interaction network, as computed on *k*>1. This is consistent with the theoretical results by Dorogovtsev and Mendes (2002), who proved that introducing a fraction of non-preferential attachment edges in evolving networks with preferential attachment does not suppress the power-law dependence of its degree distribution, but only increases the scaling exponent thereof.

This result holds when the best-fit power-law models is computed over *k*≥*k*_min_, where *k*_min_ is, as usual, the value of *k* that minimizes the Kolmogorov-Smirnov distance between the simulated in-degree distribution and its best-fit power-law model. When it is computed over the whole support of the in-degree distribution (*k*≥1), it also holds, except for *ν*_1_=1; in this case, the values of the exponents in the control and in the treatment groups are not statistically distinguishable. Tables 8 and 9 show, for each value of *ν*_1_ and *ν*_2_, the average value of the scaling parameter *α*, and the result (expressed in *p*-value) of a *t*-test on the null hypothesis that such average value is the same as the corresponding statistics in the control group, against the alternative hypothesis that the former is greater than the latter. Table 8 refers to *k*≥1, whereas Table 9 refers to *k*_min_.

**Table 8:** Average values of the power-law model’s exponent *α* in the control group and in the treatment group by values of *ν*_1_ and *ν*_2_, computed over *k*>1

Treatment groups, average *α*	*ν*_1_ = 0.0	*ν*_2_ = 0.2	*ν*_2_ = 0.4	*ν*_2_ = 0.6	*ν*_2_ = 0.8	*ν*_2_ = 1
*ν*_1_ = 0.0	3.03	3.03	3.03	3.03	3.03	3.03
*ν*_1_ = 0.2	2.87	2.87	2.88	2.87	2.87	2.87
*ν*_1_ = 0.4	2.76	2.76	2.76	2.76	2.76	2.76
*ν*_1_ = 0.6	2.67	2.67	2.67	2.67	2.66	2.66
*ν*_1_ = 0.8	2.59	2.59	2.59	2.59	2.59	2.59
*ν*_1_ = 1	2.53 (0.08)	2.53 (0.05)	2.53 (0.03)	2.53 (0.10)	2.53 (0.01)	2.53 (0.04)
Control group, average *α*: 2.52						

The number in parenthesis is the *p*-value associated to a *t*-test that *α*(*t* *r* *e* *a* *t* *m* *e* *n* *t*)=*α*(*c* *o* *n* *t* *r* *o* *l*). We only show *p*-values greater it the difference is non-significant at the 0.01 level

**Table 9:** Average values of the power-law model’s exponent *α* in the control group and in the treatment group by values of *ν*_1_ and *ν*_2_, computed over *k*≥*k*_min_

Treatment groups, average *α*	*ν*_2_ = 0.0	*ν*_2_ = 0.2	*ν*_2_ = 0.4	*ν*_2_ = 0.6	*ν*_2_ = 0.8	*ν*_2_ = 1
*ν*_1_ = 0.0	3.29	3.30	3.30	3.30	3.32	3.30
*ν*_1_ = 0.2	3.12	3.13	3.13	3.11	3.11	3.09
*ν*_1_ = 0.4	2.97	2.99	2.97	2.95	2.97	2.98
*ν*_1_ = 0.6	2.85	2.84	2.87	2.86	2.86	2.87
*ν*_1_ = 0.8	2.76	2.77	2.77	2.75	2.74	2.77
*ν*_1_ = 1	2.69	2.69	2.70	2.70	2.69	2.68
Control group, average *α*: 2.64						

We omit the *p*-values associated to a *t*-test that *α*_*treatment*_=*α*_*control*_, as they are smaller than 0.01 in all cases

### The influence of *ν*1 and *ν*2

**Fig. 5:**
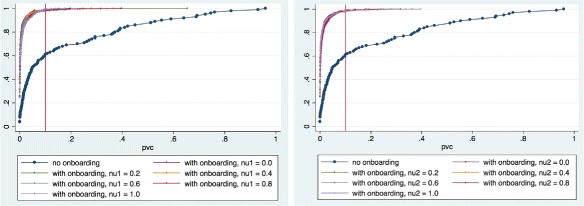
Cumulate Density Functions of *p*-values returned by goodness-of-fit tests to the (best-fit) power-law models for in-degree distributions of the interaction networks in the control and treatment groups. Sixty percent of the networks evolved without onboarding (*dark blue*) have degree distributions that test negatively for H1. When onboarding is introduced, that percentage rises to almost 100%. On the *left* figure, the treatment group interaction networks have been grouped according to the value taken by *ν*_1_; on the *right*, they have been grouped according to the value taken by *ν*_2_

We now turn to the question of the role played by *ν*_1_ and *ν*_2_ within the treatment group. Figure 5 show the cumulate density functions of the *p*-values in the control and treatment groups as *ν*_1_ and *ν*_2_ vary in the case of *k*>1.

Onboarding effectiveness *ν*_1_ and community responsiveness *ν*_2_ do not seem seem to affect the goodness-of-fit to power law of in-degree distributions much. This is clearest in Table 3, as well as in Fig. 5. This is likely to be simply an effect of the large impact of onboarding: the percentage of non-power law distributions is already close to 100% and cannot increase any further.

**Fig. 6:**
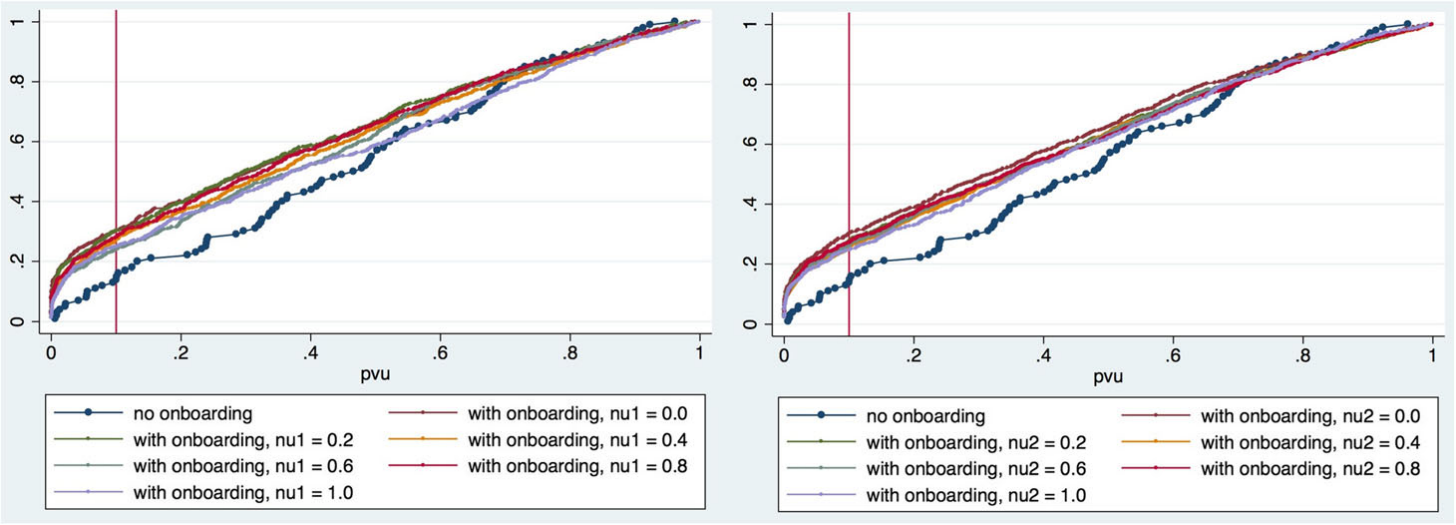
Cumulate Density Functions of *p*-values returned by goodness-of-fit tests to the (best-fit) power-law models for in-degree distributions of the interaction networks in the control and treatment groups, for *k*>*k*_*min*_. Sixteen percent of the networks evolved without onboarding (*dark blue*) have degree distributions that test negatively for H2. When onboarding is introduced, that percentage rises to 25-30%. On the *left* figure, the treatment group interaction networks have been grouped according to the value taken by *ν*_1_; on the *right*, they have been grouped according to the value taken by *ν*_2_

To dig deeper into this result, consider what happens when we allow our lower bound *k*_*min*_ to vary, so as to maximize the in-degree distribution’s goodness-of-fit to a power law model. From Table 5, we observe that the number of rejects tends to decrease as *ν*_1_ and *ν*_2_ increase. This tendency is mirrored by that of the average *p*-values in our goodness-of-fit tests to increase with both *ν*_1_ and *ν*_2_ (Table 6). Figure 6 shows this more clearly.

Intuitively, the community manager’s act of onboarding new members introduces a different law of motion into an evolving network otherwise based on preferential attachment. This shows as an increase in the number of in-degree distributions that are not a good fit for a power function. However, if the community manager is successful, her action will prompt more activity (by the newcomer, via the parameter *ν*_1_), and this extra activity does follow a preferential attachment rule. This pushes back the shape of the in-degree distribution towards the power function. In the left chart of Fig. 6, the curves representing the values of *ν*_1_ are pushed down as *ν*1 increases, towards that described by the control group.

As for *ν*_2_, we would expect it to act in the opposite direction as *ν*_1_. This is because, if the community is highly responsive (high *ν*_2_), more edges will be generated that do not follow a preferential attachment rule, but simply target the one newcomer node. This effect, however, is in practice dampened by a non-linear response of the goodness-of-fit tests with respect to additional edges targeting the newcomer. Adding the second non-preferential attachment edge does not have as much effect on the test as adding the first one. This shows up as the curves in the right part of Fig. 6, representing different values of *ν*_2_ being mashed together.

Regression analysis is unable to either confirm or falsify the intuition from Fig. 6. We generated 6 dummy variables, each taking value 1 when *ν*_1_=*c* and 0 otherwise, with *c*∈{0.0,0.2,0.4,0.6,0.8,1}; next, we generated 6 more dummy variables for the same values of *ν*_2_. We then estimated a linear regression model with the *p*-value of our goodness-of-fit test (computed for *k*>1) as the dependent variable and the 12 dummy variables as its predictors. The results are:

1. Coefficients on predictors corresponding to different values of *ν*_1_ are non-significant. The coefficient on the variable corresponding to *ν*_1_=0.4 is positive (as expected) and weakly significant (*p*-value: 0.026).
2. Coefficients on predictors corresponding to different values of *ν*_2_ are non-significant.
3. Coefficients on interaction terms between *ν*_1_ and *ν*_2_ are not significant.
4. We ran *F*-tests of joint significance of the group of predictors corresponding to different values of *ν*_1_; different values of *ν*_2_; and the interaction terms thereof. The null hypothesis of non-significance was not rejected by any of the tests.

Similar results hold when *p*-values are computed for *k*>*k*_min_.

## Discussion and conclusions

We examine data from three online communities. We find that the interaction networks in the two that are employing onboarding policies were topologically different the one that is not. We turn to the question to whether this difference might encode the mathematical signature of onboarding policy itself. To answer this, we build a time-dependent simulation model of an online community, in two (otherwise identical) versions: with and without onboarding of new members. We find that onboarding policies induce a poorer fit of power-law models to the in-degree distributions of the resulting interaction networks. This effect shows in all key parameters that describe power law models. When onboarding is enacted:

- More simulated networks fail the test of goodness-of-fit to a power law distribution. For *k*>1, almost all fail it.
- *p*-values of the best-fit power low models are lower.
- The values of *k* that minimise the Kolmogorov-Smirnov distance between the best-fit power-law models and the observed data are greater.
- Scaling parameters are greater: onboarding makes the allocation of incoming edges more equal.

Furthermore, we find that varying our onboarding effectiveness (*ν*_1_) and community responsiveness (*ν*_2_) does not have a large impact on the outcome of the simulation.

We next turn to a discussion of these results, and their potential for real-world application.

### Accounting for degree distribution shape in the interaction networks of online communities

Our simulation model incorporates two forces. The first one is preferential attachment; the second is onboarding. The former is meant to represent the rich-get-richer effect observed in many real-world social networks; the latter is meant to represent the onboarding action of moderators and community managers. The former’s effect is known to lead to the emergence of an in-degree distribution that approximates a power-law model. The latter’s effect is more subtle, because it is in turn composed of two effects. The first one consists in the direct action of the moderator, which always targets the newcomer; the second one in the actions that might be undertaken as a result of well-executed onboarding policy.

The direct action of the moderators creates edges pointing to nodes not selected by preferential attachment. In the model, this increases the number of community-created edges, which does target nodes selected via preferential attachment. In sum, onboarding increases connectivity; adds extra edges according to a non-preferential attachment rule; and, except in the case of *ν*_1_=*ν*_2_=0, also adds edges according to preferential attachment. Its net effect on the goodness-of-fit is hard to determine a priori; in practice *ν*_1_ and *ν*_2_ turn out to have a surprisingly small effect.

In fact, our results suggest that a highly effective community manager and a highly responsive community can drive the degree distribution closer to the power law state. This appears to reflect the generation of more edges allocated by preferential attachment as a consequence of the onboarding activity, though the differences are too small for solid statistical analysis.

The behaviour of the community manager as encoded in our simulation model accounts for different results in tests relating to Hypothesis 1 (*k*>1) and Hypothesis 2 (*k*>*k*_min_). Both in the model and in real life, onboarding always targets newcomers to online communities. By doing so, moderators hope to help shy newcomers turn into confident, active community members. This, however, does not prevent everyone else to receive incoming edges, allocated by preferential attachment. Therefore, we expect that the degree distributions generated by our model to be power law-shaped, but with power law behaviour “drowned out” by non-preferential attachment edges being created at low levels of *k*. This is indeed what we observe, in the form of a stronger rejection of Hypothesis 1 than of Hypothesis 2 (compare Tables 3 and 4 with Tables 5 and 6).

### Applications and limitations

We undertook this research work in the hope of discovering a simple empirical test that could be used to assess the presence and effectiveness of online community management policies, onboarding among them. The guiding idea is that the agency of online community managers and moderators is guided by a logic *other* than the rich-get-richer dynamics that spontaneously arises in many social networks. Such dynamics is associated to power-law shaped degree distributions, which we can regard as the default state for social interaction networks. We conjecture that enacting community management policies, such as onboarding, would result in altering the shape of the online community’s interaction network and its degree distribution. We furthermore conjecture that the precise nature of such deviations can be interpreted, and ultimately translated into statistical tests.

Our results are in accordance with the first of the two conjectures. The second, however, is only very partially confirmed.

Throughout the paper, we test in-degree distributions for goodness-of-fit to a power function. Its null hypothesis is that such distributions follow a power law. If the test does not reject the null, we conclude from the “Results” section that no onboarding is at work. If the test does reject the null, however, we cannot draw any conclusion. This result is compatible with the presence of onboarding, but also with any number of other processes that might be at work.

This is not a major concern for our purposes. The use case we have in mind for our empirical test is this: an organisation has instructed its community manager to onboard new members as they join, and wishes to assess the quality of their work. The organisation knows already which policy it is enacting; what it does not know is how well it works. Even in this case, a do-not-reject test result tells the organization that the community manager is not carrying out the work, but a reject test result cannot confirm she is, and certainly cannot assess her performance.

The goodness-of-fit test does also not tell an organisation whether the performance of their community manager and the responsiveness of their community is improving over time. Improvement in the community manager’s performance is captured by increases in *ν*_1_; improvement in community responsiveness is captured by increases in *ν*_2_. We have shown that their value does not have a detectable effect on the test.

### Directions for future research

There are several directions in which our work could be taken further. The first is a full and systematic exploration of the parameter space, with the goal of assessing our results’ robustness with respect to model specification. In this paper we restrict ourselves to the presence and effectiveness of the onboarding action in a baseline model which is closely modeled on Dorogovtsev’s and Mendes’s results (Dorogovtsev and Mendes 2002); it would be useful to test for how these results carry through as we alter other parameters of the model, such as the number of edges *m* created at each time step, and the additional attractiveness parameter *A*.

Secondly, we could attempt to make the model into a more realistic description of a real-world online community. Such an attempt would draw attention onto how some real-world phenomena, when incorporated in the model, influence its results. It would also carry the advantage of allowing online community management professionals to more easily interact with the model and critique it.

Finally, we could attempt to gauge the influence of onboarding and other community management policies on network topology by indicators other than the shape of its degree distribution, such as the presence of subcommunities.

## Endnotes

^1^ The literature on stochastic actor-oriented models goes several steps further, and models interaction in a social network assuming that all participants pursue goals with respect to their position in the network (Snijders 1996). We do not explore this direction in the present paper because such models require the assumption of invariant network size. In our context, that would be a zero-growth online community. We reject such an assumption as too unrealistic.

^2^ See http://www.innovatoripa.it

^3^ See https://edgeryders.eu

^4^ See http://community.matera-basilicata2019.it

## Appendix

### A1. Testing for goodness-of-fit of a power law distribution

The goodness-of-fit tests we employed were built following a procedure indicated by Clauset et al. (2009, pp. 15–18). What follows summarizes it in the context of the paper. The test’s null hypothesis is that the empirical data are distributed according to a power law model; the alternative hypothesis is that they are not.

First, we fit the data for the degree distribution of a network generated by our model to a discrete power-law model, using maximum likelihood estimation. When we are testing for goodness-of-fit of the entire degree distribution, we set the fitted power-law model lower bound to 1; when we are testing for goodness-of-fit of the distribution’s upper tail only, we choose a lower bound such that the Kolmogorov-Smirnov distance *D* between the power law model and the empirical data is minimized. Formally, define:

$$D = \max_{k \geq k_{\min}} | S(k) - P(k) | $$

Here, *S*(*k*) is the cumulative density function of the data for the observations with value at least *k*_min_, and *P*(*k*) is the cumulative density function for the power-law model that best fits the data in the region *k*≥*k*_min_. The value of *k*_min_ that minimizes the function *D* is the estimate for the model’s lower bound.

Next, we generate a large number of power-law distributed synthetic datasets with the same scaling parameter, standard deviation and lower bound as those of the distribution that best fits the empirical data. We fit each of these synthetic datasets to its own power-law model and calculate the *D* statistics of each one relative to its own model. Finally, we count what fraction of the values of *D* thus computed is larger than the value of *D* computed for the empirical data. This fraction is interpretable as a *p*-value: the probability that data generated by our estimated best-fit power-law model will be more distant from the model than our empirical data (“distant” in the Kolmogorov-Smirnov sense). A *p*-value close to zero indicates that it is quite unlikely that the estimated power-law model would generate empirical data so distant from the fitted power function; a *p*-value close to one, on the contrary, indicates that the estimated power model is quite likely to generate empirical data that are further away from the fitted power function than the ones we collected.

Generating artificial datasets requires a treatment for the region below *k*_min_ that differs from that of the one above it. We proceed as follows. Assume that our observed dataset has *n* observations total and *n*_*tail*_ observations such that *k*≥*k*_min_. To generate a synthetic datasets with *n* observations, we repeat the following procedure *n* times:

- With probability *n*/*n*_*tail*_ we generate a random number *k*_*i*_ with *k*_*i*_≥*k*_min_, drawn from a power law with the same scaling parameter as our best-fit model.
- Otherwise, with probability 1−*n*/*n*_*tail*_, we select one element uniformly at random from among the elements of the observed dataset in the region *k*<*k*_min_.

At the end of the process, we will have a synthetic dataset that follows the estimated power-law model for *k*≥*k*_min_, but has the same non-power law distribution below *k*_min_.

This test requires we decide how many synthetic datasets to generate for each test; and what is the threshold value below which we reject the null hypothesis. Again based on (Clauset et al. 2009) we make the following decisions:

- We set the number of artificial datasets generated to 2500. This corresponds to an accuracy of about 0.01, based on an analysis of the expected worst-case performance of the test.
- We conservatively set the rejection threshold at 0.01.

### A2. Choosing parameter values

The simulation’s computational intensity prevented us from conducting a thorough exploration of its behaviour across the whole parameter space. It follows we had to pick values from some parameters. In this section we discuss briefly our choice of parameter values. The choice of *m*=1 implies that the number of edges in the networks in our control group will be equal to the number of nodes; we initialize the network with two nodes connected by two edges (one in each direction), then add one node and one edge at each time step. A glance at Fig. 1 shows that this is unrealistic. The real-world online communities described in Section Empirical data all display a number of edges with is a multiple of the number of nodes.

We justify this choice as follows: we have no pretence at realism. Rather, we are interested in pitting against each other two phenomena, that of preferential attachment, that tends to generate rich-gets-richer dynamics; and that of onboarding, that tends to introduce a measure of equality. The way we modeled onboarding is by having one single incoming edge targeting the only newcomer to the community at each timestep; we therefore chose to have one single non-onboarding generated edge at each timestep. It seems reasonable that our choice would make these two forces roughly equivalent to each other, and make the impact of onboarding on the in-degree distribution easier to detect.

The choice of *A*=1 follows from another, and more fundamental, modelling choice. We mimic Dorogovtsev’s and Mendes’s approach, where the network being modeled is directed and the probability of a new edge to target a node with in-degree *k* is proportional to *k* (Dorogovtsev and Mendes 2002); this contrasts with Barabási’s and Albert’s approach, that models the network as undirected and assumes that the probability of a new edge to target a node is proportional to its total degree. In a Dorogovtsev-Mendes type model, new nodes have, by construction, in-degree zero, whereas in a Barabási-Albert type model new nodes have total degree one. It follows that, in a Dorogovtsev-Mendes type model, the parameter *A* tunes the “traction” of preferential attachment: the higher its value, the weaker the grip of pure preferential attachment. For *A*=0 Dorogovtsev-Mendes type models degenerate into “multiple star networks”, where the probability of newcomers to receive an edge is zero, and all edges target the nodes initially in the network for all time.

Setting *A*=1 we make the probability of a newcomer to receive its first edge equal to one half that of an incumbent participant who already has one incoming edge to receive its second one, one third of that of an incumbent participant who already has two incoming edges to receive its third one and so on. One can check that this behaviour mimics that of the simplest, and best known, Barabási-Albert type model.

## References

[CR1] Barabasi AL (2005). The origin of bursts and heavy tails in human dynamics. Nature.

[CR2] Barabási AL, Albert R (1999). Emergence of scaling in random networks. Science.

[CR3] Barabási AL, Albert R, Jeong H (1999). Mean-field theory for scale-free random networks. Physica A Stat Mech Appl.

[CR4] Borgatti SP, Mehra A, Brass DJ, Labianca G (2009). Network analysis in the social sciences. Science.

[CR5] Burt RS (2009). Structural Holes: The Social Structure of Competition.

[CR6] Clauset A, Shalizi CR, Newman ME (2009). Power-law distributions in empirical data. SIAM Rev.

[CR7] Cottica, A, Melançon G, Renoust B (2016) Testing for the signature of policy in online communities In: International Workshop on Complex Networks and Their Applications, 41–54.. Springer.

[CR8] De Liddo A, Sándor Á, Shum SB (2012). Contested collective intelligence: Rationale, technologies, and a human-machine annotation study. Comput Supported Coop Work (CSCW).

[CR9] Diplaris, S, Sonnenbichler A, Kaczanowski T, Mylonas P, Scherp A, Janik M, Papadopoulos S, Ovelgoenne M, Kompatsiaris Y (2011) Emerging, Collective Intelligence for Personal, Organisational and Social Use. In: Bessis N Xhafa F (eds), 527–573.. Springer, Berlin.

[CR10] Dorogovtsev SN, Mendes JFF (2002). Evolution of networks. Adv Phys.

[CR11] Grabowicz PA, Aiello LM, Eguíluz VM, Jaimes A (2013). Distinguishing topical and social groups based on common identity and bond theory. Proceedings of the Sixth ACM International Conference on Web Search and Data Mining.

[CR12] Hodas NO, Lerman K (2014). The simple rules of social contagion. Sci Rep.

[CR13] Java A, Song X, Finin T, Tseng B (2007). Why we twitter: understanding microblogging usage and communities. Proceedings of the 9th WebKDD and 1st SNA-KDD 2007 Workshop on Web Mining and Social Network Analysis.

[CR14] Klein M (2012). Enabling large-scale deliberation using attention-mediation metrics. Computer Supported Cooperative Work (CSCW).

[CR15] Kraut RE, Resnick P, Kiesler S, Burke M, Chen Y, Kittur N, Konstan J, Ren Y, Riedl J (2012). Building Successful Online Communities: Evidence-based Social Design.

[CR16] Kunegis J, Blattner M, Moser C (2013). Preferential attachment in online networks: measurement and explanations. Proceedings of the 5th Annual ACM Web Science Conference.

[CR17] Laniado D, Tasso R, Volkovich Y, Kaltenbrunner A (2011). When the wikipedians talk: Network and tree structure of wikipedia discussion pages. ICWSM.

[CR18] Leskovec, J, Horvitz E (2008) Planetary-scale views on a large instant-messaging network In: Proceedings of the 17th International Conference on World Wide Web, 915–924.. ACM.

[CR19] Lewis K, Kaufman J, Gonzalez M, Wimmer A, Christakis N (2008). Tastes, ties, and time: A new social network dataset using facebook.com. Soc Networks.

[CR20] Nick, B (2013) Toward a better understanding of evolving social networks. PhD thesis.

[CR21] Pierre L (1997). Collective intelligence: Mankinds emerging world in cyberspace.

[CR22] Rheingold H (1993). The Virtual Community: Homesteading on the Electronic Frontier.

[CR23] Shirky C (2008). Here Comes Everybody: The Power of Organizing Without Organizations.

[CR24] Shum SB (2003). The roots of computer supported argument visualization. Visualizing Argumentation.

[CR25] Slegg, J (2014) Facebook News Feed Algorithm Change Reduces Visibility of Page Updates. http://searchenginewatch.com/sew/news/2324814/facebook-news-feed-algorithm-tweak-reduces-visibility-of-page-updates. Accessed 13 Aug 2017.

[CR26] Snijders TA (1996). Stochastic actor-oriented models for network change. J Math Socio.

[CR27] Tapscott D, Williams AD (2008). Wikinomics: How Mass Collaboration Changes Everything.

[CR28] Zanetti MS, Sarigol E, Scholtes I, Tessone CJ, Schweitzer F, Jones AV (2012). A quantitative study of social organisation in open source software communities. 2012 Imperial College Computing Student Workshop.

[CR29] Zhang J, Ackerman MS, Adamic L (2007). Expertise networks in online communities: structure and algorithms. Proceedings of the 16th International Conference on World Wide Web.

